# Delayed neuronal cell death in brainstem after transient brainstem ischemia in gerbils

**DOI:** 10.1186/1471-2202-11-115

**Published:** 2010-09-14

**Authors:** Fang Cao, Ryuji Hata, Pengxiang Zhu, Shoichiro Takeda, Tadashi Yoshida, Nobuhiro Hakuba, Masahiro Sakanaka, Kiyofumi Gyo

**Affiliations:** 1 Department of Functional Histology, Ehime University Graduate School of Medicine, Shitsukawa, Toon, Ehime 791-0295, Japan; 2 Department of Otolaryngology, Ehime University Graduate School of Medicine, Shitsukawa, Toon-shi, Ehime 791-0295, Japan

## Abstract

**Background:**

Because of the lack of reproducible brainstem ischemia models in rodents, the temporal profile of ischemic lesions in the brainstem after transient brainstem ischemia has not been evaluated intensively. Previously, we produced a reproducible brainstem ischemia model of Mongolian gerbils. Here, we showed the temporal profile of ischemic lesions after transient brainstem ischemia.

**Results:**

Brainstem ischemia was produced by occlusion of the bilateral vertebral arteries just before their entry into the transverse foramina of the cervical vertebrae of Mongolian gerbils. Animals were subjected to brainstem ischemia for 15 min, and then reperfused for 0 d (just after ischemia), 1 d, 3 d and 7 d (n = 4 in each group). Sham-operated animals (n = 4) were used as control. After deep anesthesia, the gerbils were perfused with fixative for immunohistochemical investigation. Ischemic lesions were detected by immunostaining for microtubule-associated protein 2 (MAP2). Just after 15-min brainstem ischemia, ischemic lesions were detected in the lateral vestibular nucleus and the ventral part of the spinal trigeminal nucleus, and these ischemic lesions disappeared one day after reperfusion in all animals examined. However, 3 days and 7 days after reperfusion, ischemic lesions appeared again and clusters of ionized calcium-binding adapter molecule-1(IBA-1)-positive cells were detected in the same areas in all animals.

**Conclusion:**

These results suggest that delayed neuronal cell death took place in the brainstem after transient brainstem ischemia in gerbils.

## Background

In the central nervous system, certain areas are selectively damaged even after a brief ischemic insult, and this topographical heterogeneity is known as "selective vulnerability of the brain". Hippocampal CA1 and neocortical III, V, and VI are extremely vulnerable to ischemia and hypoxia [1]. The mechanism responsible for this vulnerability is of particular importance to establish therapeutic procedures, because elucidation of the mechanism may lead to the development of novel therapy to ameliorate ischemic damage.

Pathologic aspects and the topographic distribution of ischemic lesions after transient ischemia have been extensively studied in the rodent forebrain [2,3]. However, little is known about the distribution of ischemic lesions after transient brainstem ischemia because of the lack of reproducible brainstem ischemia models in rodents. Previously, we established a brainstem ischemia model in gerbils by occlusion of the bilateral vertebral arteries, and demonstrated selective vulnerability after permanent brainstem ischemia [4]. This gerbil model has the following advantages: (1) it produces brainstem ischemia without intracranial injury, (2) it produces severe, reproducible brainstem ischemia, and (3) it allows reperfusion.

In the present study, using this animal model, we investigated the temporal profile of ischemic lesions in the brainstem after transient brainstem ischemia in gerbils. We demonstrated ischemic lesions by immunostaining for microtubule-associated protein 2 (MAP2) in the lateral vestibular nucleus and the ventral part of the spinal trigeminal nucleus three days after transient brainstem ischemia, while these ischemic lesions were not found one day after ischemia. This delayed neuronal damage in the brainstem is reminiscent of the delayed neuronal cell death in the hippocampus after transient forebrain ischemia [5].

## Methods

### Animals and surgical procedure

Adult 12-16 week-old male Mongolian gerbils, weighing 60-80 g, were used in this study. All experiments were approved by the Ethics Committee of Ehime University Graduate School of Medicine and were conducted according to the Guidelines for Animal Experimentation at Ehime University Graduate School of Medicine. The gerbils were housed in an animal room with a temperature of 21 to 23°C and a 12-hour light/dark cycle (light on: 7 a.m. to 7 p.m.). The animals were allowed free access to food and water until the end of the experiment.

The gerbils were randomly divided into four groups, which were subjected to brainstem ischemia for 15 min and reperfused for 0 d (just after ischemia), 1 d, 3 d and 7 d (n = 4 in each group). Sham-operated animals (n = 4) were used as control. Animals were anesthetized with 1% halothane in 70% N_2_O and 30% O_2_. Anesthetized animals were orotracheally intubated with a ventilation tube. To facilitate access to the vertebral arteries, animals were placed in the supine position on a table tilted at approximately 30° to the horizontal. An anterior midline cervical incision was made, and the musculi longus colli were dissected to expose the vertebral arteries just before their entry into the transverse foramina of the cervical vertebrae. Both vertebral arteries were looped with 4-0 silk sutures. Then, the suture around each vertebral artery was pulled by a 5-g weight to occlude the circulation for 15 min. Consequently, apnea was observed within 1 min after occlusion, and subsequent convulsions were observed in all four limbs for about 1 min. After convulsions had ceased, all animals became unresponsive and lost their corneal reflex. Mechanical ventilation was initiated immediately after apnea was elicited during ischemia. The tidal volume was set to 1 ml and the rate was set to 70 breaths per minute. After 15 min of ischemia, the sutures were cut and removed to allow recirculation, which was confirmed by visual observation through an operating microscope. Within 10 min after reperfusion, spontaneous breathing reappeared and mechanical ventilation was ceased within 15 min after reperfusion.

Rectal temperature was maintained between 36.5 and 37.0°C by a heating lamp and a heating pad connected to a thermistor (ATB-1100, Nihon Koden, Tokyo, Japan) during surgery and until 1 h after reperfusion. After resuscitation, the animals were maintained in an air-conditioned room at about 22°C.

### Histological procedures

After deep anesthesia with a lethal dose of sodium pentobarbital (0.1 g/kg), the gerbils were perfused with 4% paraformaldehyde in 0.1 M phosphate buffer (pH 7.4) and the brain was dissected out. After fixation with the same fixative for overnight the brain was dehydrated and embedded in paraffin. To investigate the temporal profile of ischemic lesions in the brainstem, we performed immunostaining for MAP2, IBA-1 and GFAP at the level of the lateral vestibular nucleus in the brainstem (5.5 mm caudal to the bregma) since this area has been reported to be most vulnerable to ischemia [4]. Coronal 5-μm-thick sections were examined by immunostaining for microtubule-associated protein 2 (MAP2), IBA-1 and glial fibrillary acidic protein (GFAP). Sections were immunostained using a Vectastain ABC Elite Kit (Vector Laboratories; Burlingame, Calif) with polyclonal anti-MAP2 (donated by Dr. Niinobe, Osaka University), polyclonal anti-IBA-1 (019-19741, Wako, Osaka, Japan) or monoclonal anti-GFAP (G9369, Sigma, St. Louis, USA) antibodies. Endogenous peroxidase in deparaffinized tissue sections was blocked for 10 minutes with 3% H_2_O_2 _in deionized water, followed by blocking with 10% goat serum diluted in 0.2% Tween-20 in phosphate buffered saline at room temperature for 1 hour. The tissues were then incubated with primary antibody (anti-MAP2, 1:1000; anti-IBA-1, 1:500; anti-GFAP, 1:500) at 4°C overnight. Tissue sections were washed and incubated with secondary antibody (1:1000) for 1 hour at room temperature. After washing, sections were incubated with ABC complex for 30 minutes at room temperature, and then stained with the chromogenic substrate 3, 3-diaminobenzidine tetrahydrochloride (DAB) and H_2_O_2_, until optimal staining was obtained.

### Measurement of ischemic lesions

MAP2-stained sections were analyzed and images were viewed using a microscope (ECLIPSE E800, Nikon, Tokyo, Japan). The ischemic lesions detected by the loss of immunoreaction for MAP2 were measured and quantification was performed on images using ImageJ software (National Institutes of Health, Bethesda, MD).

### Statistics

All values are given as mean ± SD. Statistical analysis was performed with the Statistical Package for the Social Sciences, release 15 (SPSS ver. 15). Differences were analyzed using one-way ANOVA followed by Bonferroni's multiple comparison test. A p value of less than 0.05 was considered to indicate statistical significance.

## Results

### Immunohistochemical investigation

Four gerbils each were used for the reperfusion periods of 0, 1 d, and 3 d. As for the reperfusion period of 7 d, we evaluated three animals because one animal died of respiratory failure 5 days after ischemia. Sham-operated animals (n = 4) were used as control. Loss of immunoreaction for MAP2 in neuropils, nerve cell bodies, and dendrites was used as the criterion for the presence of ischemic lesions. The findings were compared with those in sham-operated controls. Each brain section was examined by two investigators; and whenever there was any uncertainty, a third investigator examined the specimen without any prior information.

### Just after brainstem ischemia

**Figure 1:**
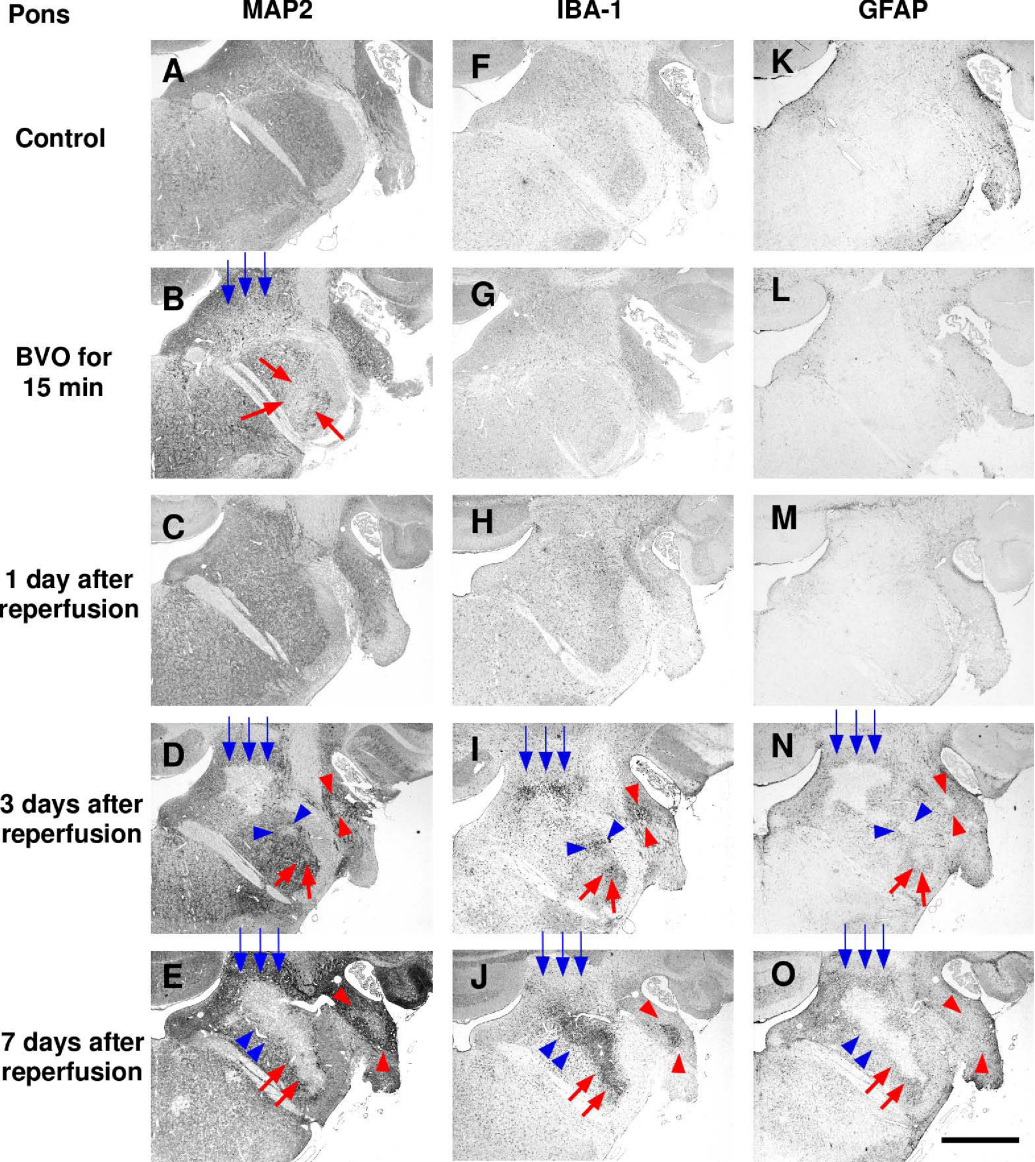
**Representative photomicrographs of immunostaining after transient brainstem ischemia for 15 min (pons)**. Left column shows immunoreactivity for MAP2 (A-E); middle column shows immunoreactivity for IBA-1 (F-J); right column shows immunoreactivity for GFAP (K-O). Ischemic lesions with loss of MAP2 staining were detected in the lateral vestibular nucleus (LVe: B; blue arrows) and the ventral part of the spinal trigeminal nucleus (Sp5: B; red arrows) after bilateral vertebral artery occlusion (BVO) for 15 min. These ischemic lesions had disappeared at 1 day after reperfusion (C). At 3 days and 7 days after reperfusion, ischemic lesions appeared again and expanded further (D and E; blue and red arrows). New ischemic lesions were detected in the dorsal part of Sp5 (D and E; blue arrowheads) and ventral cochlear nucleus (VC) (D and E; red arrowheads). At the same time, clusters of amoeboid microglia/macrophages were detected in the same areas (I and J; blue and red arrows/arrowheads). At 3 days and 7 days after reperfusion, immunoreactivity for GFAP was lost in the ischemic lesions, and increased immunoreactivity for GFAP was detected around ischemic lesions (N and O; blue and red arrows/arrowheads). Scale bar = 1 mm.

**Figure 2:**
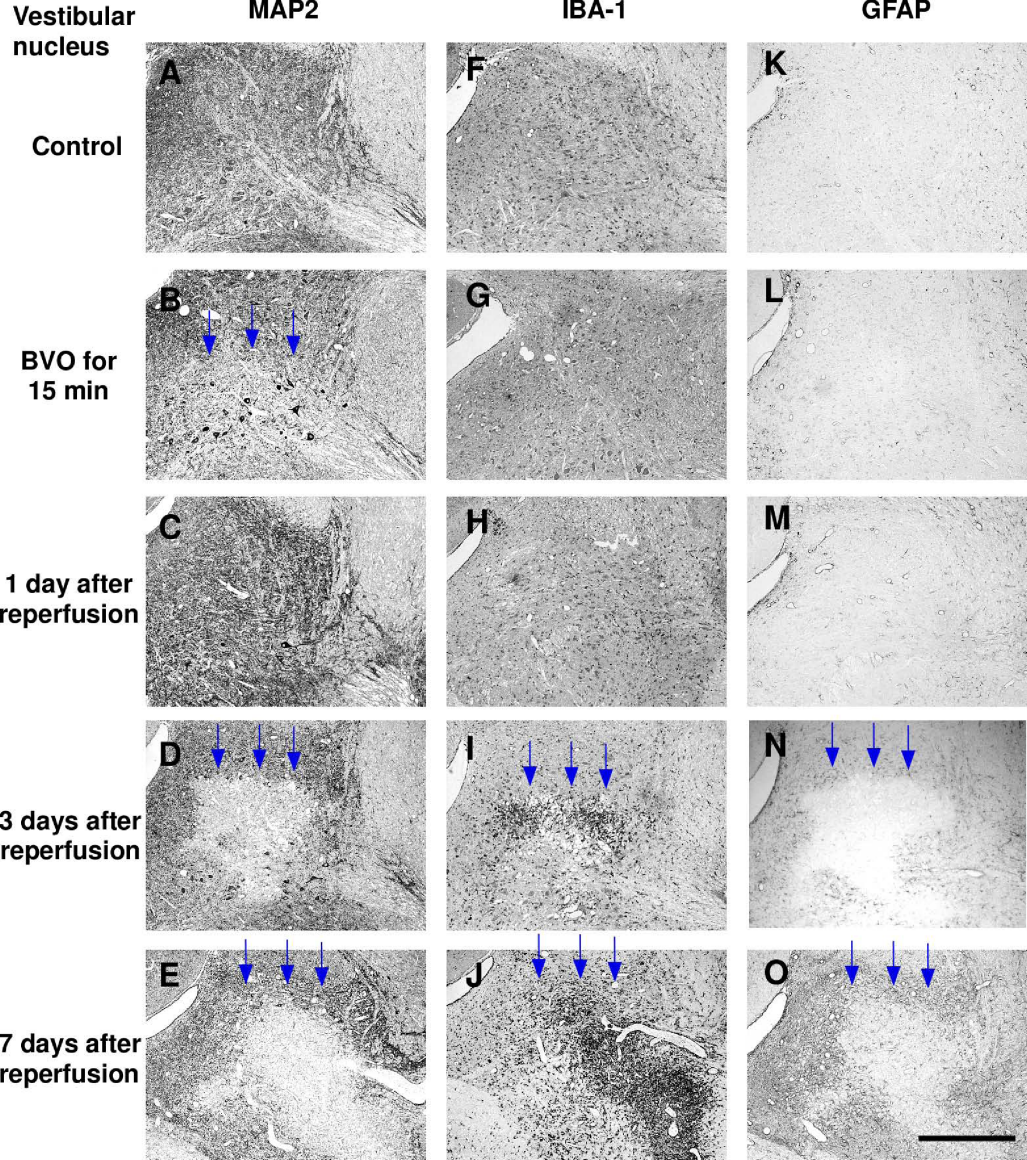
**Representative photomicrographs of immunostaining after transient brainstem ischemia for 15 min (vestibular nucleus)**. Left column shows immunoreactivity for MAP2 (A-E); middle column shows immunoreactivity for IBA-1 (F-J); right column shows immunoreactivity for GFAP (K-O). Ischemic lesions with loss of immunoreactivity for MAP2 were seen in the lateral vestibular nucleus after bilateral vertebral artery occlusion (BVO) for 15 min (B; blue arrows), and these lesions had disappeared at 1 day after reperfusion (C). However, ischemic lesions had reappeared and expanded further at 3 days and 7 days after reperfusion (D and E; blue arrows). Clusters of IBA-1-positive amoeboid microglia/macrophages (I and J; blue arrows) and loss of expression of GFAP (N and O; blue arrows) were detected in the same areas where MAP2 expression was markedly lost at 3 days and 7 days after reperfusion. Increased immunoreactivity for GFAP (N and O; blue arrows) was also detected around ischemic lesions at 3 days and 7 days after reperfusion. Scale bars = 0.5 mm.

**Figure 3:**
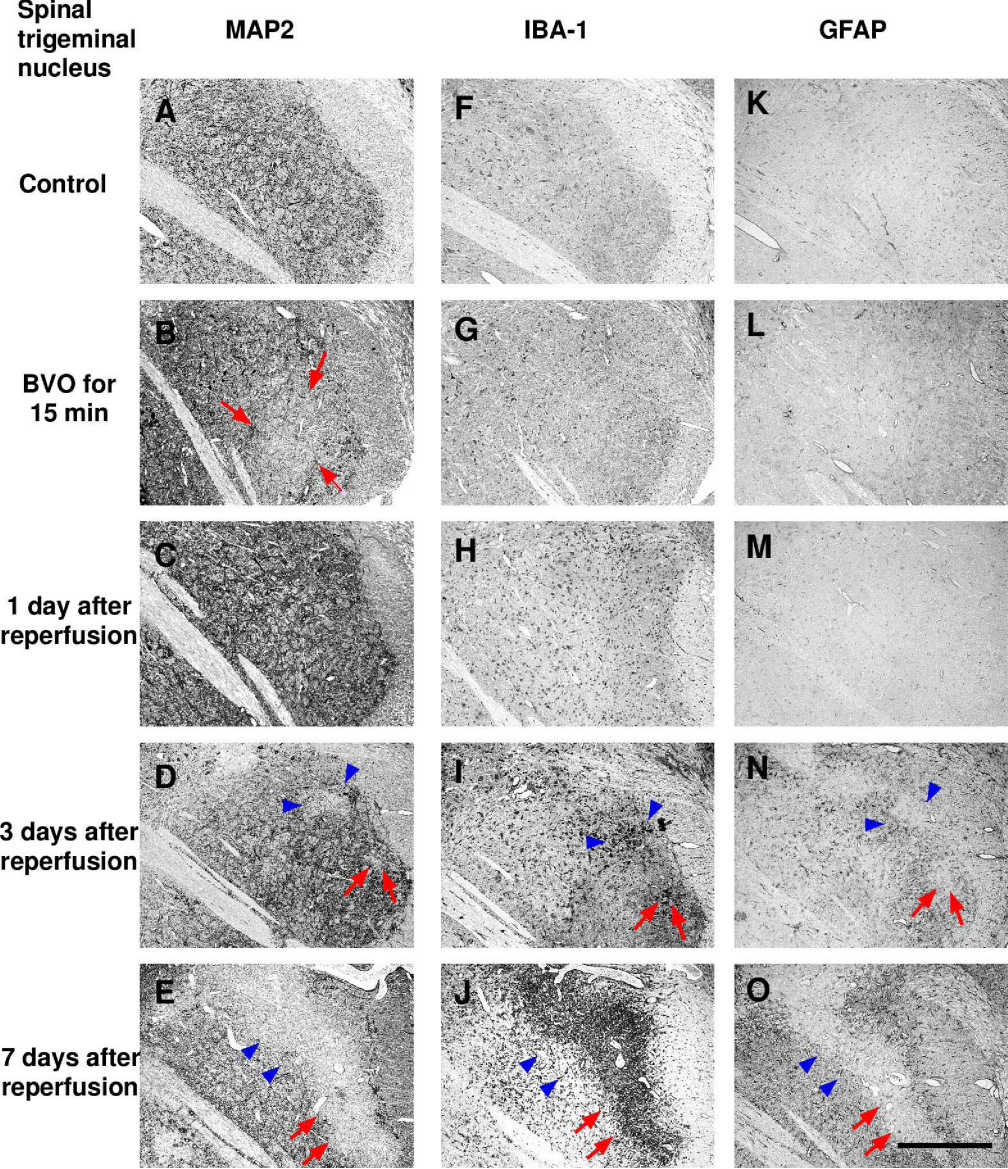
**Representative photomicrographs of immunostaining after transient brainstem ischemia for 15 min (spinal trigeminal nucleus)**. Left column shows immunoreactivity for MAP2 (A-E); middle column shows immunoreactivity for IBA-1 (F-J); right column shows immunoreactivity for GFAP (K-O). Ischemic lesions with loss of immunoreactivity for MAP2 were seen in the ventral part of Sp5 after bilateral vertebral artery occlusion (BVO) for 15 min (B; red arrows), and these lesions had disappeared at 1 day after reperfusion (C). However, ischemic lesions had reappeared and expanded further (D and E; red arrows) and new ischemic lesions were detected in the dorsal part of Sp5 (D and E; blue arrowheads) at 3 days and 7 days after reperfusion. Clusters of IBA-1-positive amoeboid microglia/macrophages (I and J; red arrows and blue arrowheads) and loss of GFAP expression (N and O; red arrows and blue arrowheads) were detected in the same areas where MAP2 expression was lost at 3 days and 7 days after reperfusion. Increased immunoreactivity for GFAP (N and O; red arrows and blue arrowheads) was also detected around ischemic lesions at 3 days and 7 days after reperfusion. Scale bars = 0.5 mm.

Ischemic lesions detected by immunostaining for MAP2 were found in the lateral vestibular nucleus (LVe; blue arrows in Figure 1B) and the ventral part of the spinal trigeminal nucleus (Sp5; red arrows in Figure 1B) in all 4 animals (100%). Higher magnification photomicrographs of ischemic lesions showed loss of immunoreaction for MAP2 in neuropils and nerve cell bodies in LVe (blue arrows in Figure 2B) and the ventral part of Sp5 (red arrows in Figure 3B). Compared with sham-operated controls, there was no change in IBA-1 (a marker of microglia and monocytic lineage) and GFAP (a marker of astrocytes) expression (Figure 1G and 1L).

### One day after brainstem ischemia

No ischemic lesion was detected by MAP2 staining (Figure 1C). Furthermore, there was no change in IBA-1 and GFAP expression, compared with that in sham-operated controls (Figure 1H and 1M).

### Three days after brainstem ischemia

**Figure 4:**
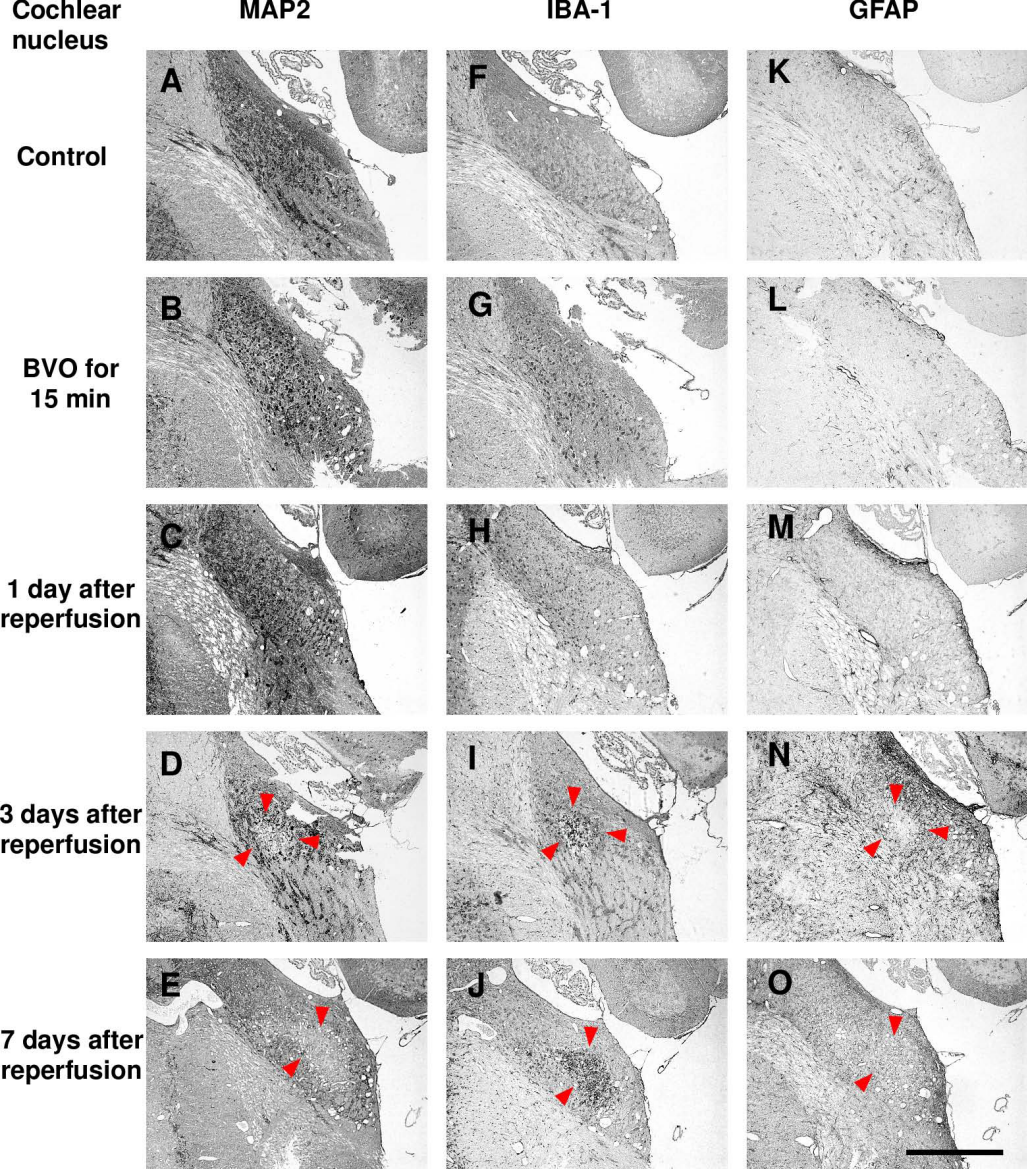
**Representative photomicrographs of immunostaining after transient brainstem ischemia for 15 min (cochlear nucleus)**. Left column shows immunoreactivity for MAP2 (A-E); middle column shows immunoreactivity for IBA-1 (F-J); right column shows immunoreactivity for GFAP (K-O). Ischemic lesions with loss of immunoreactivity for MAP2 were seen in the ventral cochlear nucleus (VC) at 3 days and 7 days after reperfusion (D and E; red arrowheads). Clusters of IBA-1-positive amoeboid microglia/macrophages (I and J; red arrowheads) and loss of GFAP expression (N and O; red arrowheads) were detected in the same areas where MAP2 expression was lost at 3 days and 7 days after reperfusion. Increased immunoreactivity for GFAP (N and O; red arrowheads) was also detected around ischemic lesions at 3 days and 7 days after reperfusion. Scale bars = 0.5 mm.

Ischemic lesions in LVe (blue arrows in Figures 1D and 2D) and the ventral part of Sp5 (red arrows in Figures 1D and 3D) appeared again in all 4 animals (100%). Compared with the ischemic lesions just after brainstem ischemia, ischemic lesions in LV expanded ventrally to include the spinal vestibular nucleus (SpVe) in 2 out of 4 animals (50%). De novo ischemic lesions were detected in the dorsal part of Sp5 (blue arrowheads in Figures 1D and 3D) and ventral cochlear nucleus (VC) (red arrowheads in Figures1D and 4D) in 2 out of 4 animals (50%).

In addition, IBA-1 immunoreactivity was markedly up-regulated in the central part of the ischemic lesions where MAP2 immunostaining was lost. Up-regulation of IBA-1 immunoreactivity was detected in LVe (blue arrows in Figures 1I and 2I) and the ventral part of Sp5 (red arrows in Figures 1I and 3I) in 3 out of 4 animals (75%). Up-regulation of IBA-1 immunoreactivity was also detected in the dorsal part of Sp5 (blue arrowheads in Figures 1I and 3I) and ventral cochlear nucleus (VC) (red arrowheads in Figures 1I and 4I) in 2 out of 4 animals (50%). Higher magnification photomicrographs demonstrated strongly IBA-1-positive cells in these areas. These IBA-1-positive cells displayed an amoeboid shape including only small perisomal lamellopodial expansions or a few unbranched processes. They were morphologically easily distinguishable from ramified microglial cells, which were recognized by their thick processes and large cell bodies.

Furthermore, immunoreactivity for GFAP disappeared in ischemic lesions where immunostaining for MAP2 was lost, whereas immunoreactivity for GFAP increased in the neighboring areas around ischemic lesions. A reduction of GFAP staining was detected in LVe (blue arrows in Figures 1N and 2N) and the ventral part of Sp5 (red arrows in Figures 1N and 3N) in 3 out of 4 animals (75%). A reduction of GFAP staining was also detected in the dorsal part of Sp5 (blue arrowheads in Figures 1N and 3N) and the ventral cochlear nucleus (VC) (red arrowheads in Figures 1N and 4N) in 2 out of 4 animals (50%). Higher magnification photomicrographs showed that GFAP-positive astrocytes were not observed in ischemic lesions where immunostaining for MAP2 was lost. Reactive astrocytes with thick, long GFAP-positive processes were distributed around ischemic lesions.

### Seven days after brainstem ischemia

**Figure 5:**
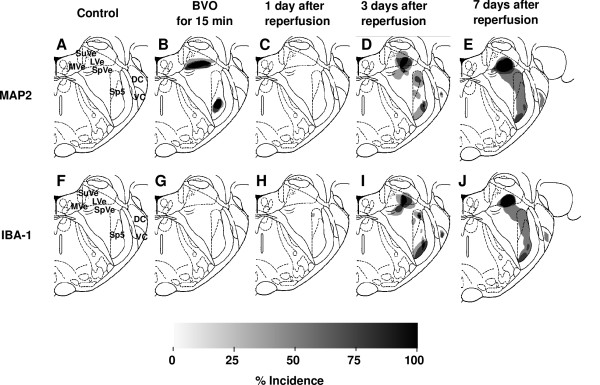
**Incidence maps of immunoreactivity for MAP2 (decrease) and IBA-1(increase) in coronal gerbil brain sections at various times after transient brainstem ischemia for 15 min (at level of pons [5.5 mm caudal to bregma])**. Areas of altered immunoreactivity were outlined in all animals examined and superimposed to represent the incidence map (%). For details, see text.

Ischemic lesions detected by immunostaining for MAP2 expanded further (Figure 5A-D). Ischemic lesions in LVe (blue arrows in Figures 1E and 2E) and the ventral part of Sp5 (red arrows in Figures 1E and 3E) appeared in all 3 animals (100%). Ischemic lesions were also detected in the dorsal part of Sp5 (blue arrowheads in Figures 1E and 3E) and the ventral cochlear nucleus (VC) (red arrowheads in Figures 1E and 4E) in 1 out of 3 animals (33%).

IBA-1 immunoreactivity was markedly up-regulated in ischemic lesions where MAP2 immunostaining was lost. Compared with the profile of IBA-1 staining three days after brainstem ischemia, strongly IBA-1-positive cells with an amoeboid shape were distributed more peripherally in ischemic lesions as well as in the center of ischemic lesions. Up-regulation of IBA-1 immunoreactivity was detected in LVe (blue arrows in Figures 1J and 2J) and the ventral part of Sp5 (red arrows in Figures 1J and 3J) in all three animals (100%). Up-regulation of IBA-1 immunoreactivity was also detected in the dorsal part of Sp5 (blue arrowheads in Figures 1J and 3J) and ventral cochlear nucleus (VC) (red arrowheads in Figures 1J and 4J) in one out of three animals (33%).

GFAP immunoreactivity disappeared in the central part of ischemic lesions where MAP2 immunostaining was lost. Immunoreactivity for GFAP increased in the periphery of ischemic lesions as well as the neighboring areas around ischemic lesions. These results suggested that reactive astrocytes proliferated in the neighboring areas around ischemic lesions and migrated into the ischemic lesions. A reduction of GFAP staining was detected in LVe (blue arrows in Figures 1O and 2O) and the ventral part of Sp5 (red arrows in Figures 1O and 3O) in all 3 animals (100%). A reduction of GFAP staining was also detected in the dorsal part of Sp5 (blue arrowheads in Figures 1O and 3O) and the ventral cochlear nucleus (VC) (red arrowheads in Figures 1O and 4O) in 1 out of 3 animals (33%).

### Temporal profile of ischemic lesions

**Figure 6:**
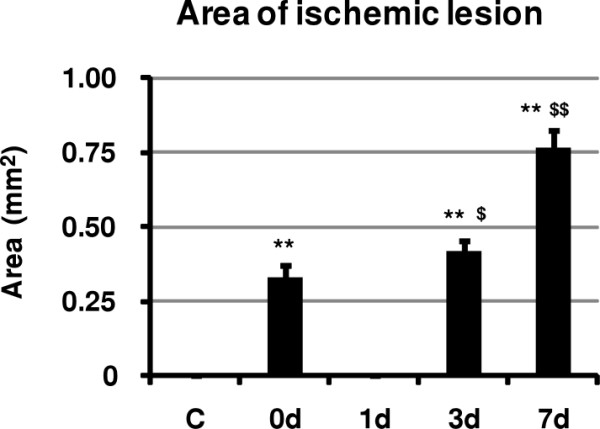
**Temporal profile of ischemic lesions detected by MAP2 staining after transient brainstem ischemia for 15 min**. The area of ischemic lesions detected by MAP2 staining in each animal was calculated. For details, see text. C: control, 0 d: just after brainstem ischemia for 15 min, 1 d, 3 d and 7 d: 1, 3 and 7 days after transient brainstem ischemia for 15 min, respectively. All values are given as mean ± SD. Differences were analyzed using one-way ANOVA followed by Bonferroni's multiple comparison test. A p value of less than 0.05 was considered to indicate statistical significance. ** and $$ indicate significant (p < 0.01) difference vs. C and 0 d, respectively. $ indicates significant (p < 0.05) difference vs. 0 d.

The total area of ischemic lesions detected by MAP2 staining in each animal was calculated and summarized in Figure 6. Just after brainstem ischemia for 15 min, the total area of ischemic lesions was 0.33 ± 0.041 [Mean ± SD] (mm^2^). Although ischemic lesions disappeared one day after brainstem ischemia, evolution of ischemic lesions was detected 3 and 7 days after transient brainstem ischemia (0.42 ± 0.034 and 0.76 ± 0.064, respectively).

## Discussion

Detection of morphological damage in early cerebral ischemia is difficult with conventional histological procedures including triphenyltetrazolium chloride and hematoxylin-eosin staining. With these conventional methods, morphological evidence of neuronal death does not become apparent until 1 to 2 hours after the onset of cerebral ischemia. However, early ischemic lesions can now be detected by applying immunohistochemical methods, and a reduction in microtubule-associated protein 2 (MAP2) immunoreactivity has been found to be an early, sensitive marker of ischemic neuronal damage [6]. In our study, by using this method, we showed that the lateral vestibular nucleus (LVe) and the ventral part of the spinal trigeminal nucleus (vSp5) were particularly vulnerable to ischemia.

In the LVe, multipolar giant neurons (Deiter's neurons) were most vulnerable to ischemia. Vestibular neurons receive excitatory glutaminergic input from the vestibular nerve [7] and commissural excitatory afferents [8]. Immunohistochemical and in situ hybridization histochemical studies revealed the highest glutamate receptor 2 (GluR2) expression in giant Deiter's neurons of the lateral vestibular nucleus and the lowest expression in small neurons throughout the vestibular nuclei [9]. These reports suggest that Deiter's neurons receive excitatory input and have selective sensitivity to excitotoxicity. As an analogy to hippocampal neurons [10], we speculate that an ischemia-induced alteration of GluR2 expression in Deiter's neurons induced cell death. Although further investigations are required to clarify the mechanisms underlying this selective vulnerability and delayed neuronal damage, they may also be related to several other factors such as the degree of cerebral hypoperfusion after reperfusion [11], inhibition of protein synthesis [12], neutrophil infiltration following reperfusion [13], free radical production [14], dysfunction of the mitochondrial shuttle system [15] or apoptosis [16].

Furthermore, we showed that the ischemic lesions in LVe and vSp5 had disappeared one day after reperfusion, but appeared again three days after reperfusion and thereafter. The observed loss of immunoreaction for MAP2 may reflect cytoskeletal breakdown, because MAP2 is involved in maintaining the structural integrity of the neuronal cytoskeleton [17]. The ischemia-induced rapid elevation of intracellular Ca^2+ ^concentration and subsequent activation of Ca^2+^-dependent phosphatases (e.g., calcineurin) and proteases (e.g., calpains) can lead to dephosphorylation and proteolytic degradation of MAP2 [18,19]. Therefore, ischemia-induced loss of immunoreaction for MAP2 is considered to be a reliable marker of neurons that are already undergoing irreversible processes in cell death [20]. However, Kitagawa et al. showed that loss of MAP2 immunostaining preceded the development of overt neuronal loss in a gerbil model of transient forebrain ischemia [6]. Our results are also consistent with this notion that MAP2 immunostaining can be used as an indicator of still viable neurons that will undergo irreversible injury only at a later time point.

We also demonstrated clusters of IBA-1-expressing cells in the ischemic lesions where MAP2 staining was lost three days after ischemia and thereafter. The rat *Iba1 *gene has been identified as a microglia-specific transcript [21]. The isolated *Iba1*clone was 0.8 kb, a rather small cDNA encoding a 17-kDa protein consisting of 147 amino acids. IBA-1 is an interferon-γ (IFN-γ)-inducible Ca^2+^-binding EF-hand protein that is encoded within the HLA class III genomic region. Expression of IBA-1 is mostly limited to the monocyte/macrophage lineage, and is augmented by cytokines such as IFN-γ. It was assumed that IBA-1 is a novel molecule involved in inflammatory responses and allograft rejection, as well as activation of macrophages [22]. In the normal brain, IBA-1 is highly expressed in resident microglial cells, but is never expressed in neurons and astrocytes [22]. After ischemia, IBA-1 is also expressed in activated resident microglial cells and infiltrating hematogenous macrophages [23,24].

Resident microglial cells rapidly became activated after ischemia. They developed amoeboid or rounded cell bodies and migrated rapidly into the ischemic lesion. For example, IBA-1 expression was rapidly up-regulated in the gerbil hippocampal CA1 region at 30 min after transient forebrain ischemia for 5 min [25]. However, microglial cells did not proliferate rapidly. Denes et al. showed that resident microglial cells exhibited intense proliferation at 48 and 72 h after transient occlusion of the middle cerebral artery (MCA) in the mouse. Average microglial cell number in the ischemic lesion did not increase significantly up to 48 h after transient ischemia [26]. We also demonstrated that a significant increase in Iba-1-positive cells was not detected in the ischemic cortex of the rat until one day after permanent MCA occlusion (MCAO), while a significant decrease in Iba-1-positive cells was detected even 2 h after permanent MCAO[27]. Furthermore, infiltrating hematogenous macrophages do not appear in the brain within one day after ischemia [24]. In this study, we did not detect clusters of IBA-1-expressing cells (i.e. topical proliferation of microglial cells) within one day after ischemia. Clusters of IBA-1-expressing cells initially appeared in the core of the ischemic lesions three days after ischemia, and these IBA-1-positive cells exhibited round cell bodies and possessed pseudopodia and thin filopodia-like processes, indicating a motile phagocytic phenotype. At seven days after ischemia, IBA-1-expressing cells with an amoeboid shape were distributed more peripherally in the ischemic lesions as well as in the core of the ischemic lesions. Based on their morphological features and the temporal profile of the distribution of IBA-1-positive cells in ischemic lesions, we speculated that these IBA-1-expressing cells were of hematopoietic origin, although we could not exclude the possibility that they were of resident microglial origin.

In addition, we showed that delayed progression of ischemic neural death took place in the brainstem. Although other morphological and biochemical investigations including electron microscopic study are required for further analysis, this delayed neuronal damage in the brainstem is reminiscent of the delayed neuronal death in the hippocampus after transient forebrain ischemia [5]. There is increasing evidence that microglial cells contribute to delayed neuronal death. Recruitment and activation of microglial cells gradually increase within the hippocampal CA1 area over 24 h after transient forebrain ischemia, before the degeneration of neurons [28]. Endangered neurons can release proinflammatory chemokines such as monocyte chemoattractant protein-1 (MCP-1/CCL2) and secondary lymphoid-tissue chemokine (SLC/CCL21). Expression of MCP-1 and SLC is increased in neurons after ischemia [29,30]. Subsequently, recruited and activated microglial cells produce inflammatory mediators, including interleukin-1β (IL-1β), tumor necrosis factor-α (TNF-α), and nitric oxide (NO), which contribute to delayed neuronal death [31]. Moreover, immunosuppressants, such as FK506, prevent microglial activation and neuronal damage after ischemia [32]. Consistent with these findings, our results also suggest that activated microglia/macrophages play a crucial role in this delayed neuronal cell death in the brainstem.

## Conclusions

In conclusion, we evaluated the evolution of ischemic lesions in the brainstem after transient brainstem ischemia in gerbils. Using immunostaining for MAP2, ischemic lesions were detected in LVe and vSp5 in all four animals. These ischemic lesions disappeared one day after reperfusion, but appeared again three days after reperfusion and thereafter in all animals examined. In addition, clusters of activated microglia/macrophages were detected in these ischemic lesions three days after ischemia and thereafter. These results suggest that delayed neuronal cell death took place in the brainstem after transient brainstem ischemia in gerbils.

## Abbreviations

SuVe: superior vestibular nucleus; MVe: medial vestibular nucleus; LVe: lateral vestibular nucleus; SpVe: spinal vestibular nucleus; DC: dorsal cochlear nucleus; VC: ventral cochlear nucleus; Sp5: spinal trigeminal nucleus; BVO: bilateral vertebral artery occlusion

## Competing interests

The authors declare that they have no competing interests.

## Authors' contributions

The original concept was by RH, MS and KG. Animal experiments were performed by ST and TY. Immunostaining was performed by FC and PZ. Evaluation of immunostaining was performed by FC, RH and NH. The manuscript was written and edited by FC and RH. All authors read and approved the final manuscript.
